# Therapeutic Simplification in COPD and Its Impact on RADAR Control: Treatment-Burden Reduction, Responder Profile and Structural–Behavioral Trajectories

**DOI:** 10.3390/jcm15134942

**Published:** 2026-06-25

**Authors:** Myriam Calle Rubio, Soha Esmaili, Iman Esmaili, Medardo Montenegro, María de la Rivera Lorenzo Andrés, Teresa Carro García, Yolanda Fernández Martín, Juan Luis Rodríguez Hermosa

**Affiliations:** 1 Pulmonology Department, Hospital Clínico San Carlos, 28040 Madrid, Spain; mcallerubio@gmail.com (M.C.R.); md.montenegrov@gmail.com (M.M.); 2 Instituto de Investigación Sanitaria del Hospital Clínico San Carlos (IdISSC), 28040 Madrid, Spain; soha@esmaili.ws; 3 Department of Medicine, School of Medicine, Universidad Complutense de Madrid, 28040 Madrid, Spain; 4 CIBER de Enfermedades Respiratorias (CIBERES), 28029 Madrid, Spain; 5 Pulmonology Department, Hospital Universitario La Zarzuela, Hospital Quirónsalud San Jose, 28023 Madrid, Spain; 6 Department of Medicine, School of Medicine, Universidad Antonio Nebrija, 28248 Madrid, Spain; 7 ISNS Data Analytics and Research, Vancouver, BC V6Z 1Y6, Canada; iman@esmaili.ws; 8 Health Centre Justicia, 28004 Madrid, Spain; 9 Health Centre Villarejo de Salvanés, 28590 Madrid, Spain; 10 Health Centre Goya, 28009 Madrid, Spain

**Keywords:** chronic obstructive pulmonary disease, single-inhaler triple therapy, therapeutic simplification, treatment burden, medication adherence, clinical control, mediation analysis

## Abstract

**Background**: Although single-inhaler triple therapy (SITT) improves COPD control, the specific structural and behavioral predictors of short-term clinical response following therapeutic simplification remain incompletely characterized. **Methods**: This prospective, multicenter observational study (N = 684) evaluated patients switching from triple therapy regimens involving multiple inhalers to SITT. A clinically meaningful response was defined as an intra-individual reduction of ≥2 points in the validated RADAR score at three months. **Results**: Therapeutic simplification reduced regimens requiring ≥4 inhalations/day from 46.1% to 14.3%, and poor behavioral adherence from 45.2% to 16.6%. Multivariable models identified an observed responder profile: higher baseline RADAR burden was the strongest predictor of improvement (aOR 2.00), whereas severe airflow limitation (FEV_1_ < 50%) attenuated the response. Exploratory mediation analysis indicated that 88.6% of the observed clinical stabilization was not explained by measured adherence changes, and may therefore also encompass unmeasured behavioral, educational or device-related factors. Patients burdened with both high complexity and poor adherence showed the highest rate of combined structural–behavioral improvement (25.0% vs. 4.7% overall). **Conclusions**: Switching from MITT to SITT was associated with reduced treatment complexity, improved adherence profiles, and short-term improvement in RADAR-defined clinical control. Patients with greater baseline RADAR burden and regimen complexity showed larger observed improvements.

## 1. Introduction

Achieving and maintaining clinical control is the primary therapeutic objective in the management of chronic obstructive pulmonary disease (COPD); however, suboptimal control remains highly prevalent and is frequently under-recognized in routine clinical practice [1,2]. Recent evidence establishes that, beyond fixed biological constraints such as severe airflow limitation, acute clinical instability is substantially driven by modifiable therapeutic factors [3]. In the present study, clinical instability is operationally defined as short-term loss of clinical control quantified by the RADAR score, which integrates symptoms, rescue medication use, exacerbation burden, and physical activity. Specifically, complex pharmacological regimens—characterized by the simultaneous use of multiple inhaler devices and high daily dosing frequencies—act as structural barriers that precipitate non-adherence and perpetuate poor clinical outcomes [4,5].

To mitigate these barriers, current clinical guidelines advocate for therapeutic simplification, prioritizing the transition to single-inhaler triple therapy (SITT) when treatment escalation or modification is indicated [6,7,8]. While the implementation of SITT has been associated with improved treatment persistence and reduced exacerbation rates [9], the structural and behavioral factors associated with short-term clinical improvement after simplification remain poorly characterized [10]. Furthermore, while existing predictive approaches have identified various clinical and biological characteristics associated with treatment response, these models typically emphasize baseline biological severity markers, largely neglecting the dynamic interplay between structural treatment burden, behavioral compliance, and the risk of imminent clinical instability [11]. Consequently, a critical knowledge gap persists regarding the clinical and behavioral correlates of treatment success following simplification. It remains unclear to what extent observed short-term improvement after switching to SITT is explained by reduced regimen complexity, measured adherence changes, or other accompanying factors [12]. Additionally, whether patients exhibiting greater baseline structural and behavioral dysfunction show larger observed improvements remains insufficiently characterized [13]. Elucidating these phenotypic determinants and their associated response patterns is imperative to optimize patient selection, overcome therapeutic inertia, and implement pragmatic stabilization strategies.

Therefore, this study was designed with three specific objectives. First, to characterize the structural simplification pattern by quantifying the immediate impact of transitioning to SITT on treatment burden and adherence categories. Second, to identify baseline determinants of clinical response and evaluate whether patients with greater baseline structural and behavioral dysfunction showed larger observed improvements after simplification. Third, to explore the extent to which measured adherence changes accounted for the association between structural burden reduction and short-term clinical improvement, and to describe structural–behavioral trajectories after switching to SITT. These constructs were used as operational descriptors of observed clinical trajectories rather than as externally validated clinical phenotypes or mechanistic entities.

## 2. Materials and Methods

### 2.1. Study Design and Participants

This study is a prospective, multicenter, observational cohort analysis conducted across primary care centers in Spain, representing the longitudinal intervention phase of a broader research program evaluating COPD clinical control [3,14]. The primary objective was to evaluate the structural and clinical impact of therapeutic simplification in the management of COPD.

The source population comprised patients with a spirometry-confirmed diagnosis of COPD (post-bronchodilator FEV_1_/FVC ratio < 0.70) aged 40 years or older, with a cumulative smoking history of at least 10 pack-years, who were clinically stable at baseline. The primary exposure criterion required patients to be transitioning from a multiple-inhaler triple therapy (MITT) regimen—strictly defined as the concurrent maintenance use of inhaled corticosteroids, long-acting beta2-agonists, and long-acting muscarinic antagonists administered via ≥2 separate devices—to single-inhaler triple therapy (SITT). The decision to transition to SITT was made by the treating physician as part of routine clinical care rather than through randomized allocation. Although the specific rationale for switching was not prospectively recorded, physician-directed simplification may have reflected clinical considerations such as symptom burden, observed non-adherence, inhaler handling difficulties, or patient preference. Participating investigators employed consecutive sampling of eligible patients presenting for routine follow-up. Patients were excluded if they presented with concomitant chronic respiratory diseases, had experienced an acute COPD exacerbation within the four weeks preceding enrollment, or lacked the capacity to complete structured functional questionnaires. A complete list of the participating centers and investigators who contributed to this study is provided in Supplementary Table S1. The flowchart illustrating participation in the study is shown in Supplementary Figure S1.

### 2.2. Procedures and Outcomes

Data were systematically acquired at baseline (T0) and at a scheduled 3-month follow-up (T1), integrating structured patient interviews, direct clinical measurements, and electronic health record extraction into a standardized electronic case report form.

Comorbidity burden was evaluated via the Charlson Comorbidity Index [15], and baseline clinical phenotypes and airflow limitation severity were classified according to standard national guidelines [8]. Structural treatment burden was dichotomized; high complexity was defined as regimens requiring ≥4 separate inhalations per day or the use of ≥3 distinct devices [3]. Additionally, overall structural burden was quantified using the TBI core, an unweighted additive index ranging from 0 to 3 points. The score was calculated by assigning one point for each of the following structural complexity factors: use of ≥3 inhaler devices, high dosing frequency defined as ≥4 inhalations per day, and baseline use of multiple-inhaler triple therapy, which was present by design in the source population.

Therapeutic adherence was objectively measured using the 10-item Test of Adherence to Inhalers (TAI), categorizing behavioral compliance as poor (≤45), intermediate (46–49), or good (≥50) [16].

The primary clinical outcome was the 3-month change in RADAR score, a validated 8-point prognostic index that objectively quantifies clinical control by integrating rescue medication utilization, acute exacerbation frequency, dyspnea severity based on the modified Medical Research Council scale, and functional physical activity [14,17,18]. A clinically meaningful response to the intervention (responder status) was operationally defined as an intra-individual reduction of ≥2 points from baseline to follow-up (ΔRADAR ≤ −2). For the purpose of longitudinal trajectory analysis, patient outcomes were operationally categorized into three trajectory categories based on the established minimal clinically important difference in the RADAR score: “Improved” (a reduction of ≥2 points), “Worsened” (an increase of ≥2 points), and “Stable” (a change ranging from −1 to +1 points).

To map structural and behavioral changes after therapeutic simplification, an exploratory descriptive construct termed combined structural–behavioral improvement was established for the purpose of this analysis. This construct was intended as an operational descriptor of concurrent improvement in structural burden and adherence, rather than as an externally validated clinical phenotype or evidence of a distinct causal mechanism. It captured the simultaneous occurrence of two discrete events between baseline and follow-up: the elimination of high structural burden (transitioning from ≥4 to <4 daily inhalations) coupled with the correction of maladaptive behavioral compliance (transitioning from a baseline TAI score ≤ 45 to a follow-up score ≥ 46).

### 2.3. Statistical Analysis

Continuous variables were summarized using means and standard deviations, while categorical variables were reported as absolute frequencies and percentages. Intra-individual longitudinal shifts in regimen complexity and adherence categories were evaluated using the McNemar–Bowker test.

To identify independent baseline determinants of clinical response and evaluate the observed responder profile, a multivariable Firth-penalized logistic regression model was constructed. This penalized likelihood approach was prospectively selected to eliminate small-sample bias and handle quasi-complete separation within sub-phenotypes. The model adjusted for baseline RADAR score, FEV_1_ < 50%, age, Charlson Comorbidity Index, baseline TAI score, and initial dosing frequency. The absence of multicollinearity among predictors was verified utilizing the Variance Inflation Factor, confirming all individual VIF values remained below the predefined threshold of 5 (Supplementary Table S2). Multiplicative interaction terms were introduced to evaluate phenotype-specific response gradients.

Furthermore, an exploratory mediation analysis was performed utilizing the R mediation package (version 4.5.1) to quantify the extent to which measured adherence changes accounted for the association between structural burden reduction and RADAR improvement. This approach decomposed the model-estimated association into a component not explained by measured adherence change and a component explained through longitudinal adherence improvement. The significance of the mediated component was estimated utilizing non-parametric bootstrap resampling with 1000 iterations.

To ensure analytical robustness and prevent potential bias, an attrition analysis was performed, confirming that the analyzed cohort remains representative of the study population (Supplementary Table S3). Comprehensive methodological details regarding temporal definitions and structural frameworks for bias mitigation are provided in the Supplementary Materials. Statistical significance was established at a two-sided alpha level of 0.05. All analyses were conducted using R statistical software, version 4.2.1.

### 2.4. Ethical Considerations

The study protocol was conducted in adherence to the Declaration of Helsinki and Good Clinical Practice guidelines. The study was conducted in accordance with the Declaration of Helsinki. All participants provided written informed consent, and the study protocol was approved by the Institutional Review Board (Ethics Committee for Clinical Research of Hospital Clínico San Carlos, Ref. 23/549-E, approved in Madrid, 5 September 2023).

## 3. Results

### 3.1. Structural Simplification and the Responder Profile

Therapeutic simplification to single-inhaler triple therapy (SITT) was associated with an immediate reconfiguration of the treatment environment, characterized by a synchronized reduction in regimen burden and improvement in adherence categories.

**Figure 1:**
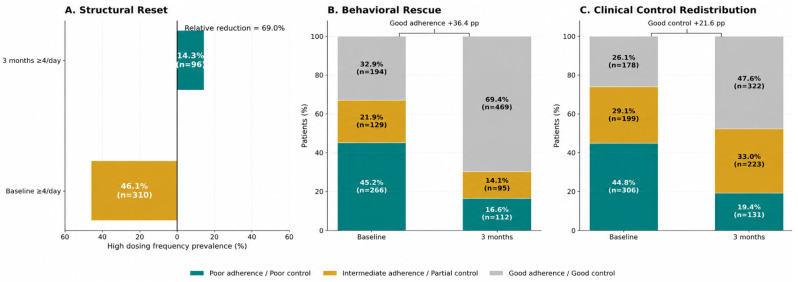
Impact of Therapeutic Simplification on Treatment Burden, Adherence Behaviors, and Overall Clinical Control. Note. Panel (**A**) displays the prevalence of high structural burden, defined as dosing frequency ≥ 4 inhalations/day, at baseline and 3 months after simplification. Panel (**B**) illustrates the redistribution of adherence categories according to the Test of Adherence to Inhalers (TAI): poor adherence (≤45 points), intermediate adherence (46–49 points), and good adherence (≥50 points). Panel (**C**) depicts the concurrent redistribution of clinical control according to RADAR score categories: poor control (≥4 points), partial control (2–3 points), and good control (0–1 points). In Panels (**B**,**C**), the same color gradient is used to represent the ordinal direction of status—unfavorable, intermediate, and favorable—rather than the same underlying variable. Percentages are calculated based on valid paired data. Statistical significance for categorical changes was determined using the McNemar–Bowker test (*p* < 0.001 for all panels).

As illustrated in Figure 1, the intervention effectively dismantled the structural complexity of the prior regimens. At baseline, 46.1% (n = 310) of the cohort required ≥4 inhalations per day; following simplification, this proportion decreased to 14.3% (n = 96), representing a 69.0% relative reduction in high-frequency dosing burden. This structural alleviation was accompanied by a marked improvement in treatment execution. Baseline prevalence of poor adherence (TAI ≤ 45) decreased from 45.2% (n = 266) to 16.6% (n = 112) at three months, while optimal adherence (TAI ≥ 50) increased to 69.4% (n = 469). Consequently, this dual structural and behavioral recovery translated into a macroscopic improvement in clinical status (Panel C in Figure 1): the proportion of patients with poor clinical control (RADAR ≥ 4) dropped from 44.8% (n = 306) to 19.4% (n = 131).

To characterize the individual profile of patients achieving clinically meaningful improvement, patients were stratified according to RADAR response status, defined as a reduction of ≥2 points. This analysis identified an observed responder profile, whereby clinical improvement was concentrated among patients with higher baseline management failure rather than disease mildness.

Table 1 shows that responders did not present with a milder biological profile; age, comorbidity burden, and severe airflow obstruction rates were comparable to non-responders. In contrast, responders were characterized by a significantly higher baseline disease burden and treatment complexity, including higher RADAR (5.18 vs. 2.07) and CAT scores (16.9 vs. 12.9) (all *p* < 0.001). Critically, responders exhibited a significantly worse behavioral baseline, with a higher prevalence of poor adherence (45.5% vs. 32.6%, *p* < 0.001). Conversely, non-responders were more likely to exhibit optimal behavior at baseline, suggesting less margin for improvement through structural simplification.

### 3.2. Independent Determinants and Phenotypic Interaction

To isolate the specific drivers of clinical response, a multivariable Firth-penalized logistic regression model was constructed to control for baseline severity and potential confounders. The full unadjusted (univariate) and adjusted (multivariable) estimates for all baseline predictors evaluated in the logistic regression model are detailed in Supplementary Table S4.

**Figure 2:**
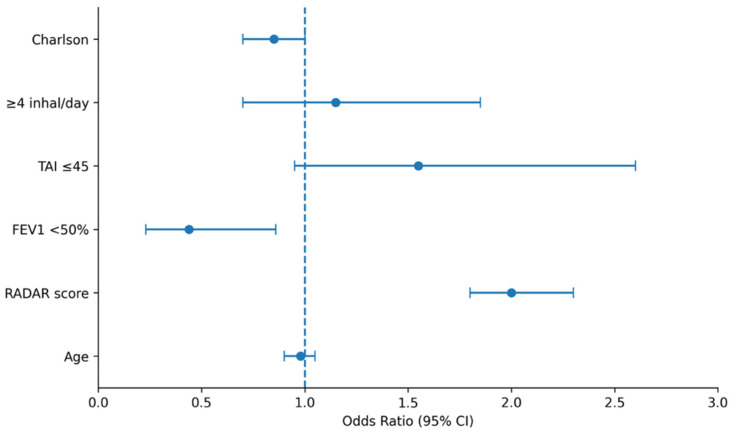
Independent Predictors of Clinical Response (Multivariable Model). Note. Forest plot displaying Adjusted Odds Ratios (aOR) and 95% Confidence Intervals from the multivariable logistic regression model (N = 684). The vertical dashed line at 1.0 represents no effect. Predictors to the right of the line indicate a higher probability of achieving the MCID in the RADAR score.

Figure 2 illustrates the dissociation between biological severity and clinical instability as predictors of success. Biological constraints acted as negative predictors: severe airflow obstruction (FEV_1_ < 50%) was independently associated with a reduced likelihood of response (aOR 0.44; 95% CI 0.23–0.86; *p* = 0.016), as were older age (aOR 0.98 per year) and higher comorbidity burden (aOR 0.85 per Charlson point). In contrast, baseline clinical instability measured by the RADAR score was the strongest positive predictor of improvement (aOR 2.00 per point; *p* < 0.001), whereas severe airflow limitation, older age, and higher comorbidity burden were associated with lower response probability. Overall, patients with higher baseline RADAR burden showed a greater probability of achieving MCID response after simplification.

While the baseline RADAR score was the strongest predictor of response, its association with clinical improvement was significantly modulated by the patient’s pre-existing behavioral–structural phenotype.

Table 2 details the interaction analysis, revealing that the efficiency of converting baseline instability into clinical success varies by regimen history. Across all phenotypes, higher baseline RADAR burden was associated with a greater probability of achieving MCID response; however, the strength of this association varied substantially by phenotype. In the reference phenotype (Low complexity + Good adherence), each one-unit increase in RADAR score doubled the odds of response (OR 2.00). This gradient was markedly amplified in patients with preserved adherence but high clinical burden (High complexity + Good), in whom each unit increase conferred a 2.55-fold increase in response probability.

A similarly steep gradient was observed in patients with poor adherence but lower clinical burden (Low complexity + Poor; OR 2.25). In contrast, patients with concordantly high clinical burden and poor adherence (High complexity + Poor Adherence) exhibited a significantly attenuated response gradient (OR 1.73), representing a 32% relative reduction in responsiveness compared with the High complexity + Good Adherence, suggesting a ceiling effect where the combination of maximal structural and behavioral barriers dampens the efficiency of the response relative to baseline severity.

**Figure 3:**
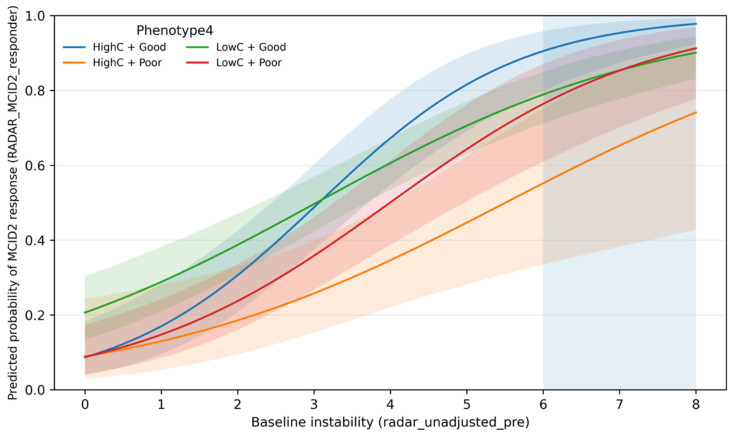
High-Yield Probability Curves: Interaction of Complexity and Adherence. Note. Probability curves derived from the interaction model. The X-axis represents the baseline RADAR score (range 0–8), and the Y-axis represents the predicted probability of achieving a clinical response (MCID). The Blue line (High Complexity + Good Adherence) demonstrates the steepest ascent, crossing the 80% probability threshold at a RADAR score of 4. The Red line (Low Complexity + Poor Adherence) and Orange line (High Complexity + Poor Adherence) show distinct trajectories, with the latter exhibiting a flatter slope at higher severity levels. Shaded areas indicate 95% confidence bands.

This non-linear interaction is visualized in the probability curves. Figure 3 presents the interaction between baseline instability (radar_unadjusted_pre) and behavioral–structural phenotype on MCID probability. The interaction was statistically significant (likelihood ratio χ^2^(3) = 16.45, *p* = 0.0009). The baseline instability slope for the High Complexity + Good Adherence phenotype was β = 0.937 (SE = 0.135), corresponding to an OR of 2.55 per unit increase. For the reference Low Complexity + Good Adherence group, the slope was β = 0.692 (OR = 2.00). The Low Complexity + Poor Adherence phenotype presented a slope of β = 0.809 (OR = 2.25). The High Complexity + Poor Adherence phenotype presented a slope of β = 0.547 (OR = 1.73), representing a 32 percent relative difference compared to the High Complexity + Good Adherence phenotype. Across other adherence mechanisms, slopes ranged from β = 0.726 to 0.804, corresponding to ORs between 2.07 and 2.23.

### 3.3. Exploratory Mediation Analysis and Clinical Trajectories

To explore whether the observed clinical improvement was associated with measured adherence changes and/or reduction in regimen complexity, an exploratory mediation analysis was performed.

Table 3 presents the exploratory decomposition of the model-estimated association. The component not explained by measured adherence change accounted for 88.6% of the total model-estimated effect. In contrast, the component explained through measured adherence improvement, while statistically significant (*p* < 0.001), accounted for 11.4% of the total effect. This pattern was consistent across alternative definitions of structural burden (range 9.5–11.4% mediated).

**Figure 4:**
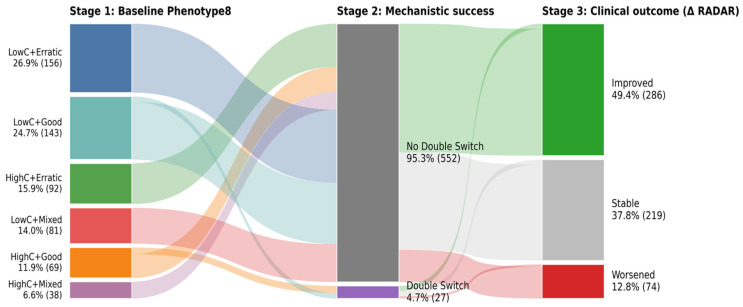
Structural–Behavioral Trajectory Roadmap. Note. Sankey diagram illustrating observed transitions from baseline behavioral–structural phenotype to clinical outcome following therapeutic simplification. Stage 1 depicts transitions from baseline phenotype to combined structural–behavioral improvement, defined as concurrent reduction in structural burden and improvement in adherence category. Stage 2 shows the downstream association between this transition pattern and clinical trajectory, categorized as Improved, Stable, or Worsened based on the change in RADAR score. Link widths are proportional to the number of patients following each pathway.

The translation of these structural and behavioral changes into individual patient trajectories is visualized in Figure 4. The diagram illustrates the redistribution of patients across the Structural–Behavioral Trajectory Roadmap, mapping transitions from baseline phenotype to the intermediate trajectory stage (combined structural–behavioral improvement), and finally to the clinical outcome (ΔRADAR).

At Stage 1, the cohort (n = 579) is distributed across six baseline phenotypes, with Low Complexity + Erratic (26.9%, n = 156) and Low Complexity + Good (24.7%, n = 143) being the most prevalent. At Stage 2, 4.7% of the total cohort (n = 27) achieved combined structural–behavioral improvement. Visual link trajectories indicate that high-complexity baseline phenotypes disproportionately contributed to this group relative to their initial prevalence (reaching 25.0% in the high complexity and poor adherence phenotype).

At Stage 3, the final clinical outcome distribution across the cohort resulted in 49.4% (n = 286) categorized as Improved, 37.8% (n = 219) as Stable, and 12.8% (n = 74) as Worsened. Downstream flow allocation demonstrates that patients achieving combined structural–behavioral improvement were primarily routed toward the Improved outcome category, presenting a visibly lower proportion of transitions to the Worsened category compared to the remainder of the cohort (95.3%, n = 552).

**Figure 5:**
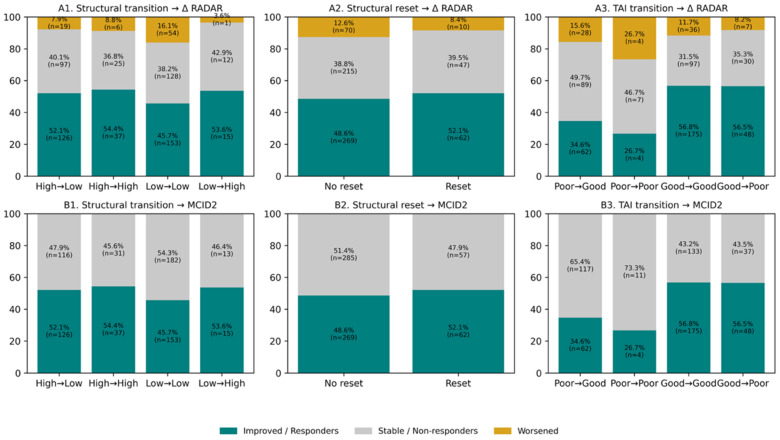
Associations of structural and behavioral transitions with clinical outcomes. Note. The figure depicts the distribution of clinical outcomes according to structural and behavioral transitions during follow-up. (**A1**–**A3**) show the proportion of patients classified as Improved, Stable, or Worsened based on the three-category RADAR change outcome. (**B1**–**B3**) display the proportion of Responders and Non-responders according to the MCID2 criterion. Structural transitions are defined by changes in regimen complexity (High → Low, High → High, Low → Low, Low → High), while behavioral transitions reflect changes in adherence status (Poor → Good, Poor → Poor, Good → Good, Good → Poor). Percentages are shown within each transition category, with absolute counts indicated in parentheses.

Figure 5 details the clinical outcomes distributed by structural and behavioral transition categories. In the dose-based structural transition analysis (ΔRADAR, Panel A1 in Figure 5), the High → Low group presented a worsening rate of 7.9%, compared to 16.1% in the Low → Low group. For the binary MCID2 outcome (Panels B1 and B2 in Figure 5), responder proportions were distributed similarly across groups, ranging from 45.7% (Low → Low) to 54.4% (High → High) in structural transitions, and from 48.6% (No simplification) to 52.1% (simplification) in the structural simplification analysis.

Regarding behavioral transitions (Panels A3 and B3 in Figure 5), the Good → Good adherence group presented an improvement rate of 56.8% and an identical MCID2 responder rate of 56.8%. Conversely, the persistently poor adherence group (Poor → Poor) recorded a 26.7% worsening rate and a 26.7% MCID2 responder rate. Patients transitioning from Poor to Good adherence achieved a 34.6% improvement rate and a 15.6% worsening rate.

## 4. Discussion

### 4.1. The Structural Simplification Pattern and Short-Term Clinical Improvement

The present study shows that the transition from multiple-inhaler regimens to single-inhaler triple therapy (SITT) was associated with structural simplification in the management of COPD. Rather than merely escalating pharmacological power, this intervention reduces structural barriers to clinical stability. As illustrated in Figure 1, the simplification process was followed by a marked contraction in high-dosing frequency and a reduction in the proportion of patients with poor adherence. Concurrently, this structural and behavioral shift was reflected in a redistribution of overall clinical control (Panel C in Figure 1), characterized by a decrease in the proportion of patients with poor clinical control. This immediate behavioral and clinical recalibration aligns with previous observations suggesting that therapeutic complexity may contribute to treatment failure [19,20]. By consolidating the pharmacological delivery system, SITT may reduce the cumulative cognitive and physical burden imposed by fragmented regimens, providing a pragmatic pathway to support short-term clinical control in a real-world primary care setting [21,22].

### 4.2. The Responder Profile in COPD Management

A central finding of this analysis is the observed responder profile. Traditionally, patients presenting with severe clinical instability, high symptom burdens, and entrenched non-adherence are perceived as refractory to intervention [23]. However, as detailed in Table 1, the responder profile was characterized precisely by this high baseline dysfunction, rather than by a milder biological disease state. Therefore, the observed pattern may reflect both clinically meaningful improvement after simplification and statistical phenomena such as regression to the mean, as these patients inherently possessed a greater baseline opportunity for improvement. The multivariable model (Figure 2) further substantiates this, revealing that while fixed biological severity—such as severe airflow obstruction (FEV_1_ < 50%)—attenuates the likelihood of a meaningful clinical response, high baseline clinical instability operates as the strongest positive predictor of improvement.

This response pattern is further illustrated by analyzing the interplay between structural burden and patient behavior (Table 2). The distinct response trajectories mapped in Figure 3 show that patients with a high structural burden coupled with adequate adherence possess the steepest probability of achieving the minimum clinically important difference. These individuals are effectively “hostages” to a complex regimen; once the structural barrier is removed, their underlying behavioral compliance may translate into greater observed clinical improvement. Conversely, the attenuated response curve observed in patients with concurrent high complexity and poor adherence suggests a ceiling effect [24], highlighting that profound behavioral dysfunction may require more comprehensive interventions beyond isolated regimen simplification [25].

### 4.3. Exploratory Mediation Findings and Combined Structural–Behavioral Improvement

The exploratory mediation analysis (Table 3) offers model-dependent insight into the extent to which measured adherence changes accounted for the association between structural burden reduction and short-term clinical improvement. Contrary to the traditional assumption that simplification improves control primarily through improved adherence [26], measured adherence improvement accounted for 11.4% of the total model-estimated effect. The remaining component should not be interpreted as evidence of a distinct structural or mechanical mechanism. Rather, it may also capture unmeasured factors accompanying therapeutic simplification, such as improved inhaler technique, device familiarity, or physician-led educational reinforcement. Prior evidence supports the plausibility that inhaler errors, inspiratory flow requirements, and device-related drug delivery may influence clinical outcomes, but these mechanisms were not directly measured in the present study [27,28].

Although at the cohort level, the component not explained by measured adherence change accounted for the largest proportion of the model-estimated association, individual patient trajectories reveal a structural–behavioral transition pattern. The flow dynamics captured in Figure 4 map structural–behavioral trajectories, specifically highlighting the concurrent reduction in structural complexity and improvement in adherence category. While this dual correction occurred in a minority of the overall cohort, it proved remarkably efficient. As highlighted in Figure 4, the specific sub-phenotype burdened with both high complexity and poor adherence exhibited a disproportionately high rate of achieving this combined improvement. These findings suggest that while profound baseline dysfunction may blunt the absolute probability of response, it may represent a concentrated opportunity for clinical improvement when simplification reduces treatment complexity and improves adherence [29]. Given the small absolute size of this subgroup, these findings are intended to support hypothesis generation and should be interpreted as an exploratory, proof-of-concept observation rather than a definitive clinical pathway. Furthermore, the analysis of specific longitudinal trajectories (Figure 5) confirms that successful transitions in either structural burden or adherence behaviors independently mitigate the risk of subsequent clinical deterioration.

### 4.4. Clinical and Conceptual Implications

These findings have practical implications for clinical decision-making and standard care algorithms. Current respiratory guidelines predominantly frame therapeutic escalation through the lens of biological treatable traits, such as eosinophilic inflammation or severe hyperinflation [29,30]. Our findings suggest that structural and behavioral features may complement this assessment by identifying potentially modifiable barriers to short-term clinical control [3,31]. The identification of high regimen complexity as an actionable barrier suggests that therapeutic simplification may represent a pragmatic strategy to improve short-term clinical control in COPD.

Recognizing the responder profile may help clinicians consider treatment burden and adherence barriers as potentially modifiable contributors to poor short-term control, while maintaining guideline-based assessment of biological treatable traits [32].

### 4.5. Limitations

This study has several methodological limitations that must be carefully considered when interpreting the findings. First, the observational, single-arm design inherently lacks a randomized control group, which precludes the absolute exclusion of natural regression to the mean or a Hawthorne effect driven by trial participation [33]. Furthermore, the specific clinical rationale for switching from MITT to SITT was physician-directed and not prospectively recorded, which introduces potential selection bias and means that residual confounding by indication cannot be definitively excluded. However, the rigorous use of intra-individual paired analyses, anchored by a previously validated minimum clinically important difference, substantially mitigates this risk by utilizing each patient as their own control. Second, the follow-up window was restricted to three months, and the primary outcome was based on change in a composite clinical score rather than hard clinical outcomes such as hospitalizations, mortality, or annualized exacerbation rates [14]. Therefore, the present findings should be interpreted as evidence of short-term improvement in RADAR-defined clinical control, while longer-term controlled studies are required to determine whether therapeutic simplification translates into sustained reductions in exacerbations, hospitalizations, or other hard clinical outcomes. Third, the exploratory mediation analysis relies on the assumption of no unmeasured confounding between the mediator (adherence) and the outcome. Given that the assumptions required for mediation analysis are difficult to fully satisfy in an observational setting, the model-estimated components should be interpreted as exploratory decompositions rather than definitive causal or mechanistic estimates. Although the model was strictly adjusted for a comprehensive array of clinical and functional covariates, residual confounding related to unmeasured socioeconomic determinants, device handling, or health literacy cannot be definitively ruled out. Moreover, several device-related factors potentially captured by the remaining model-estimated component, including inhaler technique, inspiratory flow compatibility, and drug deposition, were not directly measured. An additional limitation concerns the potential for mathematical coupling and regression to the mean, as baseline RADAR score was used as a predictor of an outcome defined by change in the same score (ΔRADAR). Accordingly, the observed responder profile should be interpreted as an exploratory response pattern, since patients with higher baseline RADAR burden had greater opportunity for improvement. Finally, the cohort consisted entirely of patients managed in primary care; thus, the extrapolation of these specific response dynamics to highly specialized tertiary care populations with end-stage biological disease should be approached with appropriate clinical caution. In addition, although RADAR has been previously validated and TBI was used as an operational measure of treatment burden, broader external validation and international use of these indices remain limited. Additionally, the structural simplification pattern, observed responder profile, and combined structural–behavioral improvement should be interpreted as descriptive operational frameworks intended to facilitate clinical interpretation of observed trajectories, rather than as validated clinical phenotypes or mechanistic constructs. Furthermore, the small sample size of the combined structural–behavioral improvement subgroup limits the robustness of subgroup-level inference.

## 5. Conclusions

In this multicenter prospective observational study, switching from MITT to SITT was associated with short-term improvements in treatment burden, adherence, and RADAR-based clinical control. Patients with higher baseline clinical burden showed larger observed improvements, although this pattern may partly reflect greater opportunity for change and regression to the mean. Exploratory trajectory and mediation analyses suggested that adherence changes explained only part of the observed improvement, but the remaining model-estimated component should not be interpreted as evidence of a specific causal mechanism. These findings support further controlled studies to determine whether therapeutic simplification improves clinically meaningful outcomes and to validate structural and behavioral response patterns in COPD.

## Figures and Tables

**Table 1:** Baseline Characteristics of Responders vs. Non-Responders.

Characteristic	Responders (MCID ≤ −2) (n = 334)	Non-Responders (n = 350)	*p*-Value
**Sociodemographic and clinical**			
Age, years (mean ± SD)	70.88 ± 10.38	71.30 ± 9.81	0.586
Female sex, n (%)	146 (43.7%)	118 (33.9%)	0.007
Active smoker, n (%)	103 (30.9%)	96 (27.4%)	0.314
BMI, kg/m^2^ (mean ± SD)	28.32 ± 9.70	28.02 ± 5.61	0.645
Pack-years (mean ± SD)	35.93 ± 23.20	38.40 ± 40.22	0.375
Charlson Comorbidity Index (mean ± SD)	2.62 ± 1.57	2.59 ± 1.88	0.844
FEV_1_ < 50%, n (%)	61 (18.3%)	49 (14.0%)	0.129
High-risk GesEPOC phenotype (Exacerbator), n (%)	223 (68.2%)	126 (36.1%)	<0.001
**Baseline disease burden**			
RADAR score (unadjusted, baseline), mean ± SD	5.18 ± 2.17	2.07 ± 2.35	<0.001
CAT score (baseline), mean ± SD	16.89 ± 5.66	12.85 ± 5.17	<0.001
**Treatment complexity (baseline)**			
Daily inhalations ≥ 4/day, n (%)	125 (54.8%)	186 (41.7%)	0.001
Devices ≥ 3, n (%)	76 (33.6%)	116 (25.7%)	0.030
TBI core (mean ± SD)	1.32± 1.37	1.06 ± 0.91	0.013
**Adherence and behavior (baseline)**			
TAI total score (mean ± SD)	43.32 ± 6.52	45.21 ± 6.32	<0.001
Poor adherence (TAI ≤ 45), n (%)	152 (45.5%)	114 (32.6%)	<0.001

Note. Data are presented as mean ± standard deviation for continuous variables and n (%) for categorical variables. Responders were defined as patients achieving a reduction of ≥2 points, corresponding to the minimal clinically important difference (MCID), in RADAR score at 3 months. TAI indicates Test of Adherence to Inhalers (range 12–60), with lower scores indicating worse adherence. High dosing frequency was defined as ≥4 inhalations per day. TBI core (Therapeutic Burden Index) is a composite score ranging from 0 to 3, calculated by assigning one point for each of the following structural complexity factors: use of multiple inhaler devices (≥3 devices), multiple-inhaler triple therapy (MITT), and high dosing frequency (≥4 inhalations per day). GesEPOC phenotypes were classified according to Spanish COPD guideline criteria. All analyses were performed in patients with available RADAR follow-up data (N = 684).

**Table 2:** Phenotype-Specific Response Gradients (Interaction Analysis).

Behavioral–Structural Phenotype	aOR per +1 (Slope)	95% Confidence Interval	*p*-Value
Low Complexity + Good Adherence (Reference)	2.00	1.68–2.37	<0.001
Low Complexity + Poor Adherence	2.25	1.71–2.95	<0.001
High Complexity + Good Adherence	2.55	1.96–3.32	<0.001
High Complexity + Poor Adherence	1.73	1.25–2.39	0.001

Note. Values represent the phenotype-specific adjusted Odds Ratio (Slope) for achieving clinical response per 1-unit increase in baseline RADAR score, derived from the Firth-penalized logistic regression interaction model. A higher aOR indicates a steeper response curve (higher marginal gain per unit of severity). The interaction term (RADAR × Phenotype) was statistically significant (Likelihood Ratio Test χ^2^ = 16.45, *p* < 0.001).

**Table 3:** Exploratory Decomposition of the Model-Estimated Association.

Component/Estimate	Coefficient (B)	Standard Error (SE)	95% Confidence Interval	Proportion Explained (%)	*p*-Value
Total Model-Estimated Association (c)	−0.044	0.010	−0.064 to −0.024	100%	<0.001
Component Not Explained by Measured Adherence Change (c′)	−0.049	0.010	−0.069 to −0.029	88.6%	<0.001
Component Explained Through Measured Adherence Improvement (ab)	0.005	0.001	0.003 to 0.007	11.4%	<0.001

Note. The model provides an exploratory decomposition of the association linking structural burden (Exposure: Core Therapeutic Burden Index) to clinical improvement (Outcome: ΔRADAR). Total Model-Estimated Association (c): the overall model-estimated association between structural burden reduction and clinical control. Component Not Explained by Measured Adherence Change (c′): the portion of the model-estimated association not accounted for by measured adherence changes. Component Explained Through Measured Adherence Improvement (ab): the portion of the model-estimated association accounted for by improvement in measured adherence. Coefficients are unstandardized. The mediated component was estimated using bootstrap resampling (1000 iterations).

## Data Availability

The dataset is available on request from the authors.

## Supplementary Materials

The following supporting information can be downloaded at: https://www.mdpi.com/article/10.3390/jcm15134942/s1, Figure S1: Flowchart of Study Participation; Table S1: Investigating Centers and Researchers; Table S2: Collinearity Diagnostics for Multivariable Predictors; Table S3: Missing Data Analysis: Comparison of Baseline Characteristics Between Included and Excluded Patients; Table S4: Full Univariate and Multivariable Logistic Regression Models Predicting Clinical Response.

## Abbreviations

The following abbreviations are used in this manuscript:

SITT	Single-inhaler triple therapy
MITT	Multiple-inhaler triple therapy
COPD	Chronic obstructive pulmonary disease
TAI	Test of Adherence to Inhalers
TBI	Therapeutic Burden Index
MCID	Minimal clinically important difference
