# Supplementary material for: Therapeutic Simplification in COPD and Its Impact on RADAR Control: Treatment-Burden Reduction, Responder Profile and Structural–Behavioral Trajectories
Source: J Clin Med. 2026 Jun 25;15(13):4942. doi: 10.3390/jcm15134942 (PMC13361885; doi:10.3390/jcm15134942)
Supplement: Supplementary file 1 [file jcm-15-04942-s001.zip › jcm-4353555-supplementary.pdf]

Supplementary Figure S1. Flowchart of Study Participation

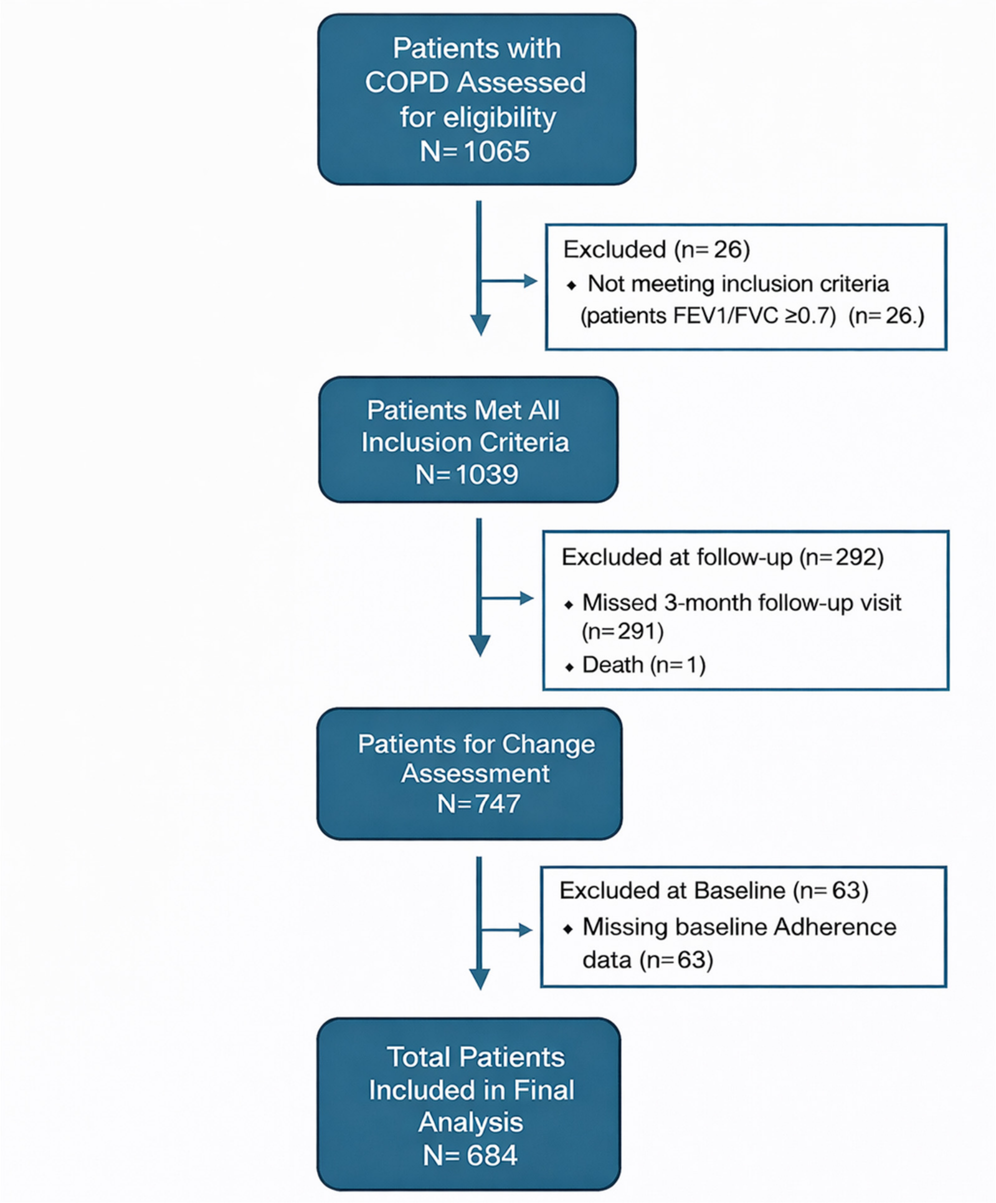

Supplementary Table S1. Investigating Centers and Researchers

The Study Group investigators are as follows:

Ana Maria , Abad Esteban , C.S. Los Yébenes , Servicio Madrileño De Salud (SERMAS).
Maria , Abad Martinez , C.S. Joaquín Rodrigo , Servicio Madrileño De Salud (SERMAS).
Alejandro , Abon Santos , C.S. Lucero , Servicio Madrileño De Salud (SERMAS).
Maria , Agudo Retuerta , C.S. Las Margaritas , Servicio Madrileño De Salud (SERMAS).
Francisco , Agüera Font , C.S. Collado Villalba Pueblo , Servicio Madrileño De Salud (SERMAS).
Lourdes , Aguilar Tejero , C.S. Londres , Servicio Madrileño De Salud (SERMAS).
Carlos , Aguilera Collado , C.S. Buitrago Lozoya , Servicio Madrileño De Salud (SERMAS).
Isabel , Alba Gago , C.S. Collado Villalba Pueblo , Servicio Madrileño De Salud (SERMAS).
Ana Maria , Alba Navas , C.S. Morata De Tajuña , Servicio Madrileño De Salud (SERMAS).
Jairo , Alegre Moreno , C.S. Dos De Mayo , Servicio Madrileño De Salud (SERMAS).
Gisella , Alfaro Leon , C.S. Dr. Luengo Rodríguez , Servicio Madrileño De Salud (SERMAS).
Lucia , Allega Gnecco , C.S. Parque Coimbra , Servicio Madrileño De Salud (SERMAS).
Yulki , Alvarez Aragoneses , C.S. Arroyomolinos , Servicio Madrileño De Salud (SERMAS).
Sonia , Alvarez Cambronero , C.S. Villarejo De Salvanés , Servicio Madrileño De Salud (SERMAS).
Sara , Alvarez De Prado , C.S. Guayaba , Servicio Madrileño De Salud (SERMAS).
Juliana , Alzate Gallego , C.S. Pavones , Servicio Madrileño De Salud (SERMAS).
Sunsi , Andres Valero , C.S. El Soto , Servicio Madrileño De Salud (SERMAS).
Maria , Andreu Tobar , C.S. San Fermín , Servicio Madrileño De Salud (SERMAS).
Maria Carmen , Anton Sanz , C.S. Villalba Estación , Servicio Madrileño De Salud (SERMAS).
Irina , Arañoz Segui , C.S. Getafe Norte , Servicio Madrileño De Salud (SERMAS).
Sonia , Arenas Gonzalez , C.S. Barcelona , Servicio Madrileño De Salud (SERMAS).
Julia , Arenas Ormeño , C.S. General Ricardos , Servicio Madrileño De Salud (SERMAS).
Ana , Arreche Artajo , C.S. Navas Del Rey , Servicio Madrileño De Salud (SERMAS).
Esther , Arrojo Arias , C.S. Maqueda , Servicio Madrileño De Salud (SERMAS).
Antonio , Arroyo Pérez , Centro De Salud Gregorio Marañón , Servicio Madrileño De Salud (SERMAS).
José Miguel , Artica Garcia , C.S. Sector Iii , Servicio Madrileño De Salud (SERMAS).
Almudena , Barahona Fernández , C.S. Valdezarza-Sur , Servicio Madrileño De Salud (SERMAS).
Alberto , Barbado Marquez , C.S. Villa De Vallecas , Servicio Madrileño De Salud (SERMAS).
Tania , Barranco Blanco , C.S. Villa De Vallecas , Servicio Madrileño De Salud (SERMAS).
Maria , Barrientos Montero , C.S. Sierra De Guadarrama , Servicio Madrileño De Salud (SERMAS).
Lorena , Beatobe Carreño , C.S. Pelayos De La Presa , Servicio Madrileño De Salud (SERMAS).
Gema , Bermejo Rubio , C.S. Arganda Felicidad , Servicio Madrileño De Salud (SERMAS).
Francisco , Bernal Hertfelder , C.S. Paracuellos De Jarama , Servicio Madrileño De Salud (SERMAS).
Gabriela , Bollati Delclos , C.S. Barcelona , Servicio Madrileño De Salud (SERMAS).
Lucia , Britoromán , C.S. San Fermín , Servicio Madrileño De Salud (SERMAS).
Jorge , Buenouceda , Consultorio De Villamanta , Servicio Madrileño De Salud (SERMAS).
David , Cabañas Moreno , C.S. General Ricardos , Servicio Madrileño De Salud (SERMAS).
David , Cabañasmoreno , C.S. General Ricardos , Servicio Madrileño De Salud (SERMAS).
Pinela , Calderin Morales , C.S. Ciudades , Servicio Madrileño De Salud (SERMAS).
Alba , Calle Armendariz , C.S. Villaviciosa De Odón , Servicio Madrileño De Salud (SERMAS).
Ricardo , Calzadilla Contrera , C.S. Nuestra Señora De Fátima , Servicio Madrileño De Salud (SERMAS).
Paula , Cañal Martinez , C.S. Villaviciosa De Odón , Servicio Madrileño De Salud (SERMAS).
Maria , Carames Garcia , C.S. Rafael Alberti , Servicio Madrileño De Salud (SERMAS).
Maria , Cardenas Joyanes , C.S. María Auxiliadora , Servicio Madrileño De Salud (SERMAS).
Teresa , Carro Garcia , C.S. Villarejo De Salvanes , Servicio Madrileño De Salud (SERMAS).
Laura , Carvajal Gonzalez , C.S. Abrantes , Servicio Madrileño De Salud (SERMAS).
Teresa , Casaseca Calvo , C.S. Puerta Del Ángel , Servicio Madrileño De Salud (SERMAS).
Almudena , Castaño Reguillo , C.S. Los Ángeles , Servicio Madrileño De Salud (SERMAS).

Carmen , Castillo De Castro , C.S. Maqueda , Servicio Madrileño De Salud (SERMAS).
Maria , Ceballos De Diego , C.S. Lucero , Servicio Madrileño De Salud (SERMAS).
Grazia , Cepeda Rodriguez , C.S. Abrantes , Servicio Madrileño De Salud (SERMAS).
Juan , Cervera Centenero , C.S. Los Ángeles , Servicio Madrileño De Salud (SERMAS).
Marta , Chamorro Gavela , C.S. Los Ángeles , Servicio Madrileño De Salud (SERMAS).
Nuria , Contreras Ramirez , C.S. Juan De La Cierva , Servicio Madrileño De Salud (SERMAS).
Miguel , Cortemarco , C.S. Valdebernardo En Moratalaz. , Servicio Madrileño De Salud (SERMAS).
Pedro , Cortes Merino , C.S. Los Castillos , Servicio Madrileño De Salud (SERMAS).
Jeniree , Da Mata Nunes , C.S. Doctor Trueta , Servicio Madrileño De Salud (SERMAS).
Blanca , Dahl Ridruejo , C.S. Villa De Vallecas , Servicio Madrileño De Salud (SERMAS).
Silvia , De Blas De Pablo , C.S. La Chopera , Servicio Madrileño De Salud (SERMAS).
Aranzazu , De Los Mozos Hernando , C.S. Sector Iii , Servicio Madrileño De Salud (SERMAS).
Maria Teresa , De Los Santos Macias , C.S. Navalcarneto I , Servicio Madrileño De Salud (SERMAS).
Cayetana , De Miguel De Juanes , C.S. Ventura Rodríguez , Servicio Madrileño De Salud (SERMAS).
Noelia , Delgado Del Carmen , C.S. Alcorcon , Servicio Madrileño De Salud (SERMAS).
Tamara , Diaz Canales , C.S. El Espinillo , Servicio Madrileño De Salud (SERMAS).
Patricia , Diaz Casanova , C.S. Miraflores , Servicio Madrileño De Salud (SERMAS).
Veronica , Diaz Garcia , C.S. Ciudades , Servicio Madrileño De Salud (SERMAS).
José Luis , Diaz Gomez , C.S. Arganda Felicidad , Servicio Madrileño De Salud (SERMAS).
Pilar , Diaz Sanchez , C.S. San Cristóbal , Servicio Madrileño De Salud (SERMAS).
Maria , Dieguez Cervantes , C.S. El Alamo , Servicio Madrileño De Salud (SERMAS).
Elena , Diesteballarín , C.S. Espronceda. , Servicio Madrileño De Salud (SERMAS).
Elisa , Diez Saguillo , C.S. Juan De La Cierva , Servicio Madrileño De Salud (SERMAS).
Susana , Dominguez Mateos , C.S. Arroyomolinos , Servicio Madrileño De Salud (SERMAS).
Norma , Doria Carlin , C.S. Los Cármenes , Servicio Madrileño De Salud (SERMAS).
Alina , Dumbraveanu , C.S. Cerro Almodóvar , Servicio Madrileño De Salud (SERMAS).
Maria Del Mar , Duque Herraiz , C.S. Entrevías , Servicio Madrileño De Salud (SERMAS).
Elias , Ekech Mesa , C.S. Espronceda , Servicio Madrileño De Salud (SERMAS).
Moises Ambioris , Elivo Morales , C.S. Dr. Trueta , Servicio Madrileño De Salud (SERMAS).
Jaime , Escalada Oliva , C.S. Guayaba , Servicio Madrileño De Salud (SERMAS).
Helena , Escudero Lamas , C.S. Ibiza , Servicio Madrileño De Salud (SERMAS).
Juan , Espino Marquez , C.S. Almendrales , Servicio Madrileño De Salud (SERMAS).
Maria Olivia , Fernandez Diez , C.S. Campo Real , Servicio Madrileño De Salud (SERMAS).
Sara , Fernandez Fernandez , C.S. Campo De La Paloma , Servicio Madrileño De Salud (SERMAS).
Paula , Fernandez Gorostieta , C.S. Segre , Servicio Madrileño De Salud (SERMAS).
Yolanda , Fernandez Martin , C.S. Goya , Servicio Madrileño De Salud (SERMAS).
Ana , Fernandez Martinez , C.S. Puerta Bonita , Servicio Madrileño De Salud (SERMAS).
Silvia , Fernandez Martinez , C.S. Dos De Mayo , Servicio Madrileño De Salud (SERMAS).
Luis Maria , Fernandez Pacheco , C.S. Ensanche De Vallecas , Servicio Madrileño De Salud (SERMAS).
Diana , Fernandez Pacheco Vila , C.S. Legazpi , Servicio Madrileño De Salud (SERMAS).
Maria , Fernandez Revalderia , C.S. Eloy Gonzalo , Servicio Madrileño De Salud (SERMAS).
Javier , Fernandez Revilla , C.S. Justicia , Servicio Madrileño De Salud (SERMAS).
José Antonio , Fernandez Ruiz , C.S. Nuestra Señora De Fátima , Servicio Madrileño De Salud (SERMAS).
Frank Raul , Fernándezmorales , C.S. Las Calesas , Servicio Madrileño De Salud (SERMAS).
Luis , Fernandez-Pacheco Corchado , C.S. Ensanche De Vallecas , Servicio Madrileño De Salud (SERMAS).
Carolina , Ferre Sanchez , C.S. Dos De Mayo , Servicio Madrileño De Salud (SERMAS).
José. , Ferrer Garcia Borrás , C.S. Almendrales , Servicio Madrileño De Salud (SERMAS).
Cristina , Fonseca Ramiro , C.S. El Soto , Servicio Madrileño De Salud (SERMAS).
Monica , Fuster Tozer , C.S. Estrecho De Corea , Servicio Madrileño De Salud (SERMAS).
Julia , Galindo Piqueras , C.S. Los Cármenes , Servicio Madrileño De Salud (SERMAS).
Elena , Garcia Bertolin , C.S. Justicia , Servicio Madrileño De Salud (SERMAS).
Gema , Garcia Chivato , C.S.. Marqués De La Valdavia , Servicio Madrileño De Salud (SERMAS).

Marta , Garcia Feliz , C.S. Nuestra Señora De Fátima , Servicio Madrileño De Salud (SERMAS).
Monica , Garcia Garcia , C.S. Valle Inclán , Servicio Madrileño De Salud (SERMAS).
Garcia Lara , C.S Campo Real , Servicio Madrileño De Salud (SERMAS).
Rosa Maria , Garcia Martin , C.S.Miguel Servet , Servicio Madrileño De Salud (SERMAS).
Begona , Garcia Ortega , C.S. Dos De Mayo , Servicio Madrileño De Salud (SERMAS).
Julia , Garcia Pascual , C.S. Arganda Felicidad , Servicio Madrileño De Salud (SERMAS).
David , Garcia Sanchez , C.S. Dr Luengo Rodríguez , Servicio Madrileño De Salud (SERMAS).
Luis , Garcia Sanchez Molina , C.S. Villa De Vallecas , Servicio Madrileño De Salud (SERMAS).
Beatriz , Garcia Serrano Jimenez , C.S. Los Yébenes , Servicio Madrileño De Salud (SERMAS).
Almudena , Garcia Uceda Sevilla , C.S. Nuestra Señora De Fátima , Servicio Madrileño De Salud (SERMAS).
Olga , Garcia Vallejo , C.S. Almendrales , Servicio Madrileño De Salud (SERMAS).
María , Garcíaperez , C.S. San Fermín , Servicio Madrileño De Salud (SERMAS).
Paulo , Gil Agapito , C.S. San Andrés , Servicio Madrileño De Salud (SERMAS).
Enrique , Gomez Garrido , C.S. Navalcarnero , Servicio Madrileño De Salud (SERMAS).
Begona , Gomez Pérez , C.S. Dos De Mayo , Servicio Madrileño De Salud (SERMAS).
Adrián , Gómezgómez , C.S. Valdebernardo , Servicio Madrileño De Salud (SERMAS).
Silvia , Gonzalez Alcantud , C.S. El Bercial , Servicio Madrileño De Salud (SERMAS).
Monica , Gonzalez Alvarez , C.S. Puerta Del Ángel , Servicio Madrileño De Salud (SERMAS).
Maria Teresa , Gonzalez Cantueso , C.S. Felipe Ii , Servicio Madrileño De Salud (SERMAS).
Nazaret , Gonzalez Sanchez , C.S. Sector Iii , Servicio Madrileño De Salud (SERMAS).
Ricardo , Gonzalez Tejada , C.S. San Fermín , Servicio Madrileño De Salud (SERMAS).
Maria Cristina , Gonzalo Gutierrez , C.S. Barcelona , Servicio Madrileño De Salud (SERMAS).
Matilde , Gonzalo Lazaro , C.S. Entrevías , Servicio Madrileño De Salud (SERMAS).
Maria Del Pilar , Guerrero Cabezas , C.S. Villablanca , Servicio Madrileño De Salud (SERMAS).
Irene , Guillermo España , C.S. Pacífico , Servicio Madrileño De Salud (SERMAS).
Maria Alexandra , Gullo Porco-Gallina , C.S. Barcelona , Servicio Madrileño De Salud (SERMAS).
Paloma , Gutierrez Sordo , C.S. Felipe Ii , Servicio Madrileño De Salud (SERMAS).
Laura , Hernán Pérez Torralba , C.S. Pavones , Servicio Madrileño De Salud (SERMAS).
Hernandez Agujetas , C.S. Las Olivas , Servicio Madrileño De Salud (SERMAS).
Marta , Hernandez Castan , C.S. Los Rosales , Servicio Madrileño De Salud (SERMAS).
Erika , Hernandez Melo , C.S. Navalcarnero I , Servicio Madrileño De Salud (SERMAS).
José , Herrera Diaz , Otro C.S. , Servicio Madrileño De Salud (SERMAS).
José , Herrera Díaz , C.S Alcalde Bartolomé , Servicio Madrileño De Salud (SERMAS).
Esther , Higueras Martinez , C.S. Los Cármenes , Servicio Madrileño De Salud (SERMAS).
Rocío , Horcajada Alocen , C.S. Los Rosales , Servicio Madrileño De Salud (SERMAS).
Paquita , Ibañez Garcia , C.S. Abrantes , Servicio Madrileño De Salud (SERMAS).
Jauregui Artola , Otro C.S. , Servicio Madrileño De Salud (SERMAS).
Jimenez Fernandez , C.S. Panaderias , Servicio Madrileño De Salud (SERMAS).
Azucena , Jimenez Garcia , C.S. Villa Del Prado , Servicio Madrileño De Salud (SERMAS).
Vladimir , Jimenez Gonzalez , C.S. Felipe Ii , Servicio Madrileño De Salud (SERMAS).
Ana Laura , Lafraya Puente , C.S. Dr. Luengo Rodríguez , Servicio Madrileño De Salud (SERMAS).
Lourdes , Laguna Delgado , C.S. Joaquín Rodrigo , Servicio Madrileño De Salud (SERMAS).
Rafael , Lesmes Gonzalez , C.S. Los Cármenes , Servicio Madrileño De Salud (SERMAS).
Salvador , Leyenda Gomez , C.S. Puerta Del Ángel , Servicio Madrileño De Salud (SERMAS).
Juan , Llorentemiñano , C.S. Eloy Gonzalo , Servicio Madrileño De Salud (SERMAS).
Lucia Del Carmen , Lopez Durando , C.S. Pavones , Servicio Madrileño De Salud (SERMAS).
Vera , Lopez Herrero , C.S. San Fermín , Servicio Madrileño De Salud (SERMAS).
Irene , Lopez Larrayoz , C.S. Doctor Luengo Rodríguez, , Servicio Madrileño De Salud (SERMAS).
Marta , Lopez Machado , C.S. Juan De La Cierva , Servicio Madrileño De Salud (SERMAS).
Topacio , Lopez Mena , C.S. Gregorio Marañon , Servicio Madrileño De Salud (SERMAS).
Fatima , Lopez Palomo , C.S. Villa Del Prado , Servicio Madrileño De Salud (SERMAS).
Sonia , Lopez Revuelta , C.S. Juan De La Cierva , Servicio Madrileño De Salud (SERMAS).

Enrique , Lopez Somalo , C.S. Cercedilla , Servicio Madrileño De Salud (SERMAS).
Vera , Lópezherrero , C.S. San Fermín , Servicio Madrileño De Salud (SERMAS).
Maria De La Rivera , Lorenzo Andres , C.S. Quince De Mayo , Servicio Madrileño De Salud (SERMAS).
Jesus , Lorenzo Francisco , C.S. Los Yébenes , Servicio Madrileño De Salud (SERMAS).
Marina , Losa Carrasco , C.S. Villablanca , Servicio Madrileño De Salud (SERMAS).
Maria José , Lougedo Calderon , C.S. El Reston , Servicio Madrileño De Salud (SERMAS).
Marta , Lozano Onrubia , C.S. Villa Del Prado , Servicio Madrileño De Salud (SERMAS).
Alberto , Manzanares Briega , C.S. Buenos Aires , Servicio Madrileño De Salud (SERMAS).
Ana , Marchan Martin , Consultorio Villamanta , Servicio Madrileño De Salud (SERMAS).
Miguel Angel , Maria Tablado , C.S. Perales De Tajuña , Servicio Madrileño De Salud (SERMAS).
Maria Teresa , Marin Becerra , C.S. General Ricardos , Servicio Madrileño De Salud (SERMAS).
Carlos , Martin-Fuertes Guio , C.S. Dos De Mayo, Servicio Madrileño De Salud (SERMAS).
José , Martin Sistiaga , C.S. San Fermín , Servicio Madrileño De Salud (SERMAS).
Maria Pilar , Martinez Merodio , C.S. La Paz , Servicio Madrileño De Salud (SERMAS).
Carmen , Martinez Blanco , C.S. Torito , Servicio Madrileño De Salud (SERMAS).
José Antonio , Martinez Campos , C.S. Presentación Sabio , Servicio Madrileño De Salud (SERMAS).
Luis , Martinez Lopez , C.S. Nuestra Señora De Fátima , Servicio Madrileño De Salud (SERMAS).
Martinez Martinez , C.S. De Campo Real , Servicio Madrileño De Salud (SERMAS).
Laura , Martinez Rego , C.S. Los Rosales , Servicio Madrileño De Salud (SERMAS).
Ana , Massa Achutegui , C.S. Villa Del Prado , Servicio Madrileño De Salud (SERMAS).
Teresa , Mazarro Enrique , C.S. Pedro Laín Entralgo , Servicio Madrileño De Salud (SERMAS).
Asuncion , Mena Garrido , C.S. Morata De Tajuña , Servicio Madrileño De Salud (SERMAS).
Valeria , Méndez Gutiérrez , C.S. Barcelona , Servicio Madrileño De Salud (SERMAS).
Susana , Menendez Alvarez , C.S. Aldea Del Fresno , Servicio Madrileño De Salud (SERMAS).
Myriam , Menendez Ortega , C.S. Sevilla La Nueva , Servicio Madrileño De Salud (SERMAS).
Karen , Merinogonzález , C.S. Torito , Servicio Madrileño De Salud (SERMAS).
Minguez Mena , C.S. El Molar , Servicio Madrileño De Salud (SERMAS).
Maria De La Salud , Molina Bermejo , C.S. El Soto , Servicio Madrileño De Salud (SERMAS).
Marta , Monge Bronchalo , C.S. La Paz , Servicio Madrileño De Salud (SERMAS).
Elisabet , Morenas Moreno , C.S. Arganda- Felicidad , Servicio Madrileño De Salud (SERMAS).
Barbara , Moreno Garcia , C.S. Nuestra Señora De Fátima , Servicio Madrileño De Salud (SERMAS).
Cristina , Muños Martinez De Salinas , C.S. Puerta Bonita , Servicio Madrileño De Salud (SERMAS).
Maria Estrella , Muñoz Crispulo , C.S. Felipe Ii , Servicio Madrileño De Salud (SERMAS).
Enrique , Muñoz Cruz , C.S. Pacífico , Servicio Madrileño De Salud (SERMAS).
Cristina , Muñoz Martinez De Salinas , C.S. Puerta Bonita , Servicio Madrileño De Salud (SERMAS).
Maria Del Pilar , Muñoz Molina , C.S. Los Ángeles , Servicio Madrileño De Salud (SERMAS).
Maria Aranzazu , Murciano Anton , C.S. Dr. Pedro Lain Entralgo , Servicio Madrileño De Salud (SERMAS).
Maria De Los Angeles , Navarro Arranz , C.S. Los Cármenes , Servicio Madrileño De Salud (SERMAS).
Maria Luz , Navas Hergueta , C.S. Navas Del Rey , Servicio Madrileño De Salud (SERMAS).
Monica , Nieto Villarrubia , C.S. El Espinillo , Servicio Madrileño De Salud (SERMAS).
Dante , Noboa Noboa , C.S. Entrevías , Servicio Madrileño De Salud (SERMAS).
Clara , Ochoa Ruiz , C.S. Las Olivas (Aranjuez) , Servicio Madrileño De Salud (SERMAS).
Miriam , Ordoñez Vicente , C.S. Londres , Servicio Madrileño De Salud (SERMAS).
Maria , Orozco Jimenez , C.S. Gregorio Marañon , Servicio Madrileño De Salud (SERMAS).
David , Palacios Martinez , C.S. Almendrales , Servicio Madrileño De Salud (SERMAS).
Julissa , Patiño Jimenez , C.S. Villablanca , Servicio Madrileño De Salud (SERMAS).
Santiago , Pequeño Leido C.S. Campamento Servicio Madrileño De Salud (SERMAS).
Veronica , Pérez Aradas , C.S. Las Olivas (Aranjuez) , Servicio Madrileño De Salud (SERMAS).
Maria Esperanza Almudena , Pérez Cañon , C.S. San Fermín , Servicio Madrileño De Salud (SERMAS).
Olga , Pérez Gandia , C.S. Doctor Trueta , Servicio Madrileño De Salud (SERMAS).
Javier , Pérez Gonzalez , C.S. La Rivota , Servicio Madrileño De Salud (SERMAS).
Encarnacion , Pérez Pérez , C.S. Los Castillos , Servicio Madrileño De Salud (SERMAS).

Patricia , Pérez Rodriguez , C.S. Alcalde Bartolomé González , Servicio Madrileño De Salud (SERMAS).
Carlos Luis , Pessegueiro Freitas , C.S. Quince De Mayo , Servicio Madrileño De Salud (SERMAS).
Marta , Pinedo Hoyos , C.S. Pedro Lain Entralgo , Servicio Madrileño De Salud (SERMAS).
Celia , Plaza Coya , C.S. Ángela Uriarte , Servicio Madrileño De Salud (SERMAS).
Marta , Porta Lopez Acevedo , C.S. Eloy Gonzalo , Servicio Madrileño De Salud (SERMAS).
Patricia , Privado Martinez , C.S. Primero De Mayo , Servicio Madrileño De Salud (SERMAS).
Alejandro , Puente Torres , C.S. Eloy Gonzalo , Servicio Madrileño De Salud (SERMAS).
Nerea , Pulgar Prieto , C.S. San Martín De Valdeiglesias , Servicio Madrileño De Salud (SERMAS).
Nuria , Puyo Rodriguez , C.S. La Rivota , Servicio Madrileño De Salud (SERMAS).
Maria Angeles , Quintana Bravo , C.S. Felipe Ii , Servicio Madrileño De Salud (SERMAS).
Alejandro , Rabanal Basalo , C.S. Los Yébenes , Servicio Madrileño De Salud (SERMAS).
Ruben , Ramos Ludeña , C.S. Juan De La Cierva , Servicio Madrileño De Salud (SERMAS).
Maria , Resino Rocha , C.S. Villaviciosa De Odon , Servicio Madrileño De Salud (SERMAS).
María , Resinorocha , C.S. Villaviciosa De Odón , Servicio Madrileño De Salud (SERMAS).
Ana , Rey Lopez , C.S. Los Ángeles , Servicio Madrileño De Salud (SERMAS).
Maria Guadalupe , Rincon Carmona , C.S. Rafael Alberti , Servicio Madrileño De Salud (SERMAS).
Isabel , Riopedre Martinez , C.S. Ciudades , Servicio Madrileño De Salud (SERMAS).
Paloma , Rius Fortea , C.S. Valdezarza-Sur , Servicio Madrileño De Salud (SERMAS).
Lorena , Rodriguez Alvarez , Consultorio Local Alpedrete , Servicio Madrileño De Salud (SERMAS).
Jorge , Rodriguez Reguera , C.S. Los Castillos , Servicio Madrileño De Salud (SERMAS).
Gloria , Rojo Grillo , C.S. Dos De Mayo , Servicio Madrileño De Salud (SERMAS).
Raquel , Roldan Lomba , C.S. Nuevo Baztan , Servicio Madrileño De Salud (SERMAS).
Estenia , Romero , C.S. General Ricardos , Servicio Madrileño De Salud (SERMAS).
Maria Luisa , Romero Garcia , C.S. Pavones , Servicio Madrileño De Salud (SERMAS).
Lucia , Romero Huete , C.S. Felipe Ii , Servicio Madrileño De Salud (SERMAS).
Maria Carmen , Romero Sanchez , C.S. Legazpi , Servicio Madrileño De Salud (SERMAS).
Sandra Lisset , Rondon Maldonado , C.S. Campo De La Paloma , Servicio Madrileño De Salud (SERMAS).
Alicia , Rueda Jesus , C.S. Navalcarnero , Servicio Madrileño De Salud (SERMAS).
Tania , Ruiz Molina , Consultorio Arroyomolinos , Servicio Madrileño De Salud (SERMAS).
José , Salinero Acevedo , C.S. Segovia , Servicio Madrileño De Salud (SERMAS).
Julia , San José , Consultorio De El Alamo. , Servicio Madrileño De Salud (SERMAS).
Sara , Sanchez Barreiro , C.S. General Ricardos , Servicio Madrileño De Salud (SERMAS).
Inmaculada , Sanchez Pulgarin , C.S. General Ricardos , Servicio Madrileño De Salud (SERMAS).
Estrella , Sanchez-Gamborino Del Rio , C.S. Vicente Soldevilla , Servicio Madrileño De Salud (SERMAS).
Eloina , Sandin De Vega , C.S. Ciudades , Servicio Madrileño De Salud (SERMAS).
Sanjurjo Navarro , C.S. Ciudad Jardín , Servicio Madrileño De Salud (SERMAS).
Maria Eugenia , Seguro Requejo , C.S. Los Castillos , Servicio Madrileño De Salud (SERMAS).
Isabel , Sepulveda Gomez , C.S. Martín De Vargas , Servicio Madrileño De Salud (SERMAS).
Alberto , Serrano Lopez De Las Hazas , C.S. Cerro Almodóvar , Servicio Madrileño De Salud (SERMAS).
Marta , Suarez Risueño , C.S. Eloy Gonzalo , Servicio Madrileño De Salud (SERMAS).
Natasa , Timbota , C.S. Santa Mónica (Rivas Vaciamadrid). , Servicio Madrileño De Salud (SERMAS).
Lucia , Tirado Jimenez , Consultorio Local Arroyomolinos , Servicio Madrileño De Salud (SERMAS).
Corina , Torres Barriga , C.S. Navalcarnero 1 , Servicio Madrileño De Salud (SERMAS).
Carolina , Torrijos Bravo , C.S. Joaquín Rodrigo , Servicio Madrileño De Salud (SERMAS).
Gloria , Uria Santamarina , C.S. Los Rosales , Servicio Madrileño De Salud (SERMAS).
José Maria , Valenzuela , C.S. Campo Real , Servicio Madrileño De Salud (SERMAS).
Maria Elena , Vaquero Fernandez , C.S. La Paz , Servicio Madrileño De Salud (SERMAS).
Gabriel , Vazquez Perfecto , C.S. Dos De Mayo , Servicio Madrileño De Salud (SERMAS).
Virginia , Viejogarcía , C.S. Navalcarnero , Servicio Madrileño De Salud (SERMAS).
Yu , Wang Gao , C.S. Joaquín Rodrigo , Servicio Madrileño De Salud (SERMAS).
Susana , Zafra Alonso , C.S. Campo Real , Servicio Madrileño De Salud (SERMAS).
Liliana , Zapata Mosquera , C.S. Santa Monica , Servicio Madrileño De Salud (SERMAS).

Supplementary Table S2. Collinearity Diagnostics for Multivariable Predictors.

**Variable**	**Tolerance**	**VIF**
Baseline RADAR score	0.89	1.12
FEV1 < 50% pred.	0.95	1.05
Age, years	0.84	1.18
Charlson Index	0.82	1.21
TAI Score ≤ 45	0.86	1.15
High Dosing (≥4/day)	0.90	1.10

Note. Collinearity diagnostics performed prior to multivariable modeling to evaluate the degree of correlation among independent predictor variables. VIF = Variance Inflation Factor.

Supplementary Table S3 presents the collinearity diagnostics for the covariates included in the multivariable model. The absence of severe multicollinearity is confirmed, as all individual VIF values remain strictly below the predefined threshold of 5.0, indicating that each variable contributes independent explanatory variance.

Supplementary Table S3. Missing Data Analysis: Comparison of Baseline Characteristics Between Included and Excluded Patients.

Variable	Included in Analysis (n=684)	Excluded (Missing Data) (n=63)	p-value
Age, years, Mean (SD)	71.1 (10.0)	71.01 (10.2)	0.281
Female sex, n (%)	264 (38.1)	35 (36.1)	0.492
Active smoker, n (%)	199 (29.1)	30 (30.9)	0.765
Charlson Index, Mean (SD)	2.65 (1.72)	2.78 (1.65)	0.618
FEV1 < 50% pred., n (%)	110 (16.1)	16 (16.5)	0.554
Core TBI Score, Mean (SD)	0.98 (0.94)	1.0 (0.98)	0.884
High Dosing (≥4/day), n (%)	311 (45.5)	28 (44.4)	0.907
Baseline RADAR Score, Mean (SD)	3.59 (1.85)	3.47 (1.80)	0.715

Note. Data are presented as mean (SD) or n (%). P-values were calculated using Student's t-test for continuous variables and Pearson's χ^2 test for categorical variables. Comparison groups are defined as patients with complete records included in the multivariable models versus those excluded due to missing data in one or more variables.

Supplementary Table S1 presents the missing data analysis comparing retained patients versus those excluded due to missing primary outcome data, validating the baseline characteristics summarized in Table 1. The absence of statistically significant differences across demographic or clinical domains ($p > 0.05$) confirms that the analyzed cohort remains representative of the original population.

Supplementary Table S4. Full Univariate and Multivariable Logistic Regression Models Predicting Clinical Response.

Variable	Univariate OR (95% CI)	p-value	Multivariable aOR (95% CI)	p-value
Baseline RADAR score	2.05 (1.75 to 2.40)	< 0.001	2.00 (1.68 to 2.37)	< 0.001
FEV1 < 50% pred.	0.40 (0.21 to 0.78)	0.006	0.44 (0.23 to 0.86)	0.016
Age, years	0.97 (0.95 to 0.99)	0.012	0.98 (0.96 to 0.99)	0.035
Charlson Index	0.82 (0.75 to 0.90)	< 0.001	0.85 (0.77 to 0.94)	0.002
TAI Score ≤ 45	1.65 (1.10 to 2.45)	0.015	1.55 (0.96 to 2.60)	0.075
High Dosing (≥4/day)	1.45 (1.05 to 2.05)	0.025	1.15 (0.71 to 1.85)	0.550

Note. Logistic regression analysis evaluating baseline predictors of achieving a clinically meaningful response (reduction of ≥2 points in the RADAR score) at 3 months (N = 684). OR = Odds Ratio; aOR = Adjusted Odds Ratio; CI = Confidence Interval. The multivariable model was fitted using Firth's penalized likelihood approach.

Supplementary Table S2 presents the full unadjusted and adjusted estimates from the logistic regression models. The analysis demonstrates that while baseline structural and behavioral dysfunctions show predictive value in univariate assessment, baseline clinical instability (RADAR score) remains the strongest independent predictor of clinical improvement in the adjusted model.
